# The structure of ICD-11 PTSD and complex PTSD in Lithuanian mental health services

**DOI:** 10.1080/20008198.2017.1414559

**Published:** 2018-01-11

**Authors:** Evaldas Kazlauskas, Goda Gegieckaite, Philip Hyland, Paulina Zelviene, Marylene Cloitre

**Affiliations:** a Department of Clinical and Organizational Psychology, Vilnius University, Vilnius, Lithuania; b School of Business, National College of Ireland, Dublin, Ireland; c Centre for Global Health, Trinity College Dublin, Dublin, Ireland; d National Center for PTSD, Veterans Affairs Palo Alto Health Care System, Palo Alto, CA, USA; e Department of Psychiatry and Behavioral Sciences, Stanford University, Palo Alto, CA, USA

**Keywords:** ICD-11, PTSD, complex PTSD, latent class analysis, validation, • The factorial and discriminant validity of ICD-11 proposals for PTSD and CPTSD were supported in a Lithuanian sample. • The new ICD-11 based measure of PTSD and CPTSD was used for measurement of symptoms. • ICD-11 PTSD and CPTSD appear to be clinically meaningful constructs within the Lithuanian context.

## Abstract

**Background**: The updated 11^th^ edition of International Classification of Diseases (ICD-11) is expected to be released by the WHO in 2018. Disorders specifically associated with stress will be included in a separate chapter in ICD-11, and will include a revision of ICD-10 PTSD as well as a new diagnosis of complex posttraumatic stress disorder (CPTSD). The proposed symptom structures of ICD-11 PTSD and CPTSD have been validated in several studies previously, however few studies have used the International Trauma Questionnaire (ITQ), a specific measure for ICD-11 PTSD and CPTSD. Given that ICD-11 PTSD and CPTSD diagnoses are intended to be applicable across different cultures and nations, it is important that the constructs be evaluated across diverse populations and languages.

**Objective**: Study of the psychological impact of trauma is relatively new in Lithuania, coinciding with its independence from the Soviet Union in the 1990s. Studies thus far reveal a population suffering from the effects of long-term and systematic political oppression and violence. The aim of this study was to assess the validity of the symptoms and structure of PTSD and CPTSD in a Lithuanian treatment-seeking sample as measured by the ITQ.

**Method**: A total of 280 patients from outpatient mental health centres participated in this study. PTSD and CPTSD symptoms were measured with the ITQ. We applied confirmatory factor analysis (CFA) and latent class analysis (LCA) for analysis of data.

**Results and conclusions**: Our study supported the ICD-11 factor structure of CPTSD, and a three-class model was supported in LCA analysis with a PTSD class, a CPTSD class, and a low symptom class. Findings support the factorial and discriminant validity of the ICD-11 proposals for PTSD and CPTSD in a unique clinical population.

## La estructura del tept y el tept complejo de la cie-11 en los servicios lituanos de salud mental

**Planteamiento**: Está previsto que la OMS publique la onceava versión actualizada de la Clasificación Internacional de Enfermedades (CIE-11) en 2018. Los trastornos específicamente relacionados con el estrés se incluirán en un capítulo separado en la CIE-11 e incluirán una revisión del TEPT de la CIE-10, así como un nuevo diagnóstico de trastorno de estrés postraumático complejo (TEPT-C). Las estructuras de síntomas propuestas para el TEPT y el TEPT-C de la CIE-11 han sido validadas en varios estudios previamente, sin embargo, solo unos pocos estudios han utilizado el Cuestionario Internacional de Trauma (ITQ, por sus siglas en inglés), una medida específica para el TEPT y el TEPT-C de la CIE-11. Dado que los diagnósticos de TEPT y TEPT-C de la CIE-11 pretenden aplicarse en diferentes culturas y naciones, es importante que los constructos se evalúen en diversas poblaciones e idiomas.

**Objetivos**: El estudio del impacto psicológico del trauma es relativamente nuevo en Lituania, coincidiendo con su independencia de la Unión Soviética en la década de 1990 y los estudios hasta el momento revelan una población que sufre los efectos de la opresión y la violencia política sistemáticas a largo plazo. El objetivo de este estudio fue evaluar la validez de los síntomas y la estructura del TEPT y el TEPT-C en una muestra lituana que buscaba tratamiento, medida con el ITQ.

**Método**: Participaron en este estudio un total de 280 pacientes de centros ambulatorios de salud mental. Los síntomas de TEPT y TEPT-C se midieron con el ITQ. Aplicamos el Análisis Factorial Confirmatorio (CFA, por sus siglas en inglés) y el Análisis de Clase Latente (LCA, por sus siglas en inglés) para el análisis de datos.

**Resultados y conclusiones**: Nuestro estudio apoyó la estructura de factores del TEPT-C de la CIE-11 y respaldó un modelo de tres clases en el análisis de LCA con una clase de TEPT, una clase de TEPT-C y una clase de pocos síntomas. Los hallazgos respaldan la validez factorial y discriminante de las propuestas de la CIE-11 para el TEPT y el TEPT-C en una población clínica singular.

## 立陶宛精神健康服务中ICD-11 PTSD和复杂 PTSD的结构

**背景：**更新的《第11版国际疾病分类（ICD-11）》将有望在2018由 WHO 发布。和应激有关的失调会在 ICD-11中作为独立章节出现，并会包括对 ICD-10 PTSD 的修订，和对复杂创伤后应激障碍（CPTSD）的新诊断。这个提议中的 ICD-11 PTSD 和 CPTSD 的症状结构已经在一些前人研究中得到了验证，但很少研究使用《国际创伤问卷（ITQ）》（一个关于 ICD-11 PTSD 和 CPTSD 的专门测量工具）。由于ICD-11 PTSD 和 CPTSD 的诊断是预期在不同的文化和国家中使用，所以对其结构在多样的人群和语言中进行评估是非常重要的。

**目标：**在立陶宛创伤的心理影响相关研究还相对较新。90年代立陶宛从苏联独立后就有研究发现一个群体受到长期和系统性政治压迫和暴力。本研究的目标是在立陶宛需求治疗样本中，使用 ITQ评估 PTSD 和 CPTSD 的症状和结构的有效性。

**方法：**招募自精神健康中心门诊部的280名病人参加了这项研究。PTSD 和 CPTSD 使用 ITQ 测量。我们使用验证性因子分析（CFA）和潜在类别分析（LCA）进行数据分析。

**结果和结论：**我们的研究支持了ICD-11的 CPTSD 因子结构，在 LCA 分析中得到一个三类别模型： PTSD 类别、CPTSD 类别和低症状类别。这些发现支持了ICD-11建议区分 PTSD 和 CPTSD 作为不同的临床人群，其因子和区分效度得到验证。

Following the proposals of the World Health Organization (WHO) Working Group for Disorders Specifically Associated With Stress for the 11^th^ Edition of International Classification of Diseases (ICD-11) (Maercker et al., 2013, 2013), empirical investigation for several of the newly defined, or refined, diagnoses is underway (e.g. Hyland et al., 2017; Karatzias et al., 2017; Keeley et al., 2016; Shevlin et al., 2017; Zelviene, Kazlauskas, Eimontas, & Maercker, 2017). Under the category of posttraumatic stress disorders, the ICD-11 will include two distinct disorders: PTSD and a new diagnosis of complex posttraumatic stress disorder (CPTSD). Several studies have supported the validity of the distinction, including a field study of international mental health providers which indicated that clinicians readily discriminated between the PTSD and CPTSD diagnoses (Keeley et al., 2016). The proposed symptom structure of ICD-11 CPTSD has been validated in several studies using archival data (e.g. Cloitre, Garvert, Brewin, Bryant, & Maercker, 2013; Cloitre, Garvert, Weiss, Carlson, & Bryant, 2014; Knefel, Garvert, Cloitre, & Lueger-Schuster, 2015) and there are ongoing investigations concerning the reliability and validity of a measure for ICD-11 PTSD and CPTSD (Hyland, Shelvin, Brewin et al., 2017; Karatzias et al., 2016; Murphy, Elklit, Dokkedahl, & Shevlin, 2016).

The ICD-11 PTSD diagnosis is comprised of three symptom clusters: re-experiencing (Re), avoidance (Av), and sense of threat (Th), which generally describe a fear reaction to a traumatic experience. ICD-11 CPTSD is comprised of two distinct factors, a PTSD factor comprised of Re, Av, and Th, as well as a factor that represents disturbances of self-organization (DSO), which is also comprised of three clusters, namely affective dysregulation (AD), negative self-concept (NSC), and disturbances in relationships (DR) (Maercker et al., 2013). These symptoms represent difficulties that occur pervasively and across different contexts. The diagnosis of CPTSD is expected to typically result from sustained, repeated, or multiple forms of trauma from which escape is difficult or impossible (e.g. childhood abuse, domestic violence, torture, war imprisonment) and to reflect the loss of emotional, psychological, and social resources which can occur under conditions of prolonged adversity (Maercker et al., 2013). The diagnoses are organized under the general family name of posttraumatic stress disorders and an individual can be diagnosed with one or the other disorder. Type of trauma history is expected to influence risk for one or the other disorder; the diagnosis is determined not by history but by symptom profile.

The distinction between PTSD and CPTSD is consistent with ICD-11 guidelines that diagnoses should have high clinical utility. This includes criteria, satisfied by the PTSD and CTPSD distinction, that the two symptom profiles accurately describe distinct classes of individuals and are easily discernible by clinicians in the field (Keeley et al., 2016). According to a recent review by Brewin et al. (2017), nine of 10 studies using a latent class analysis (LCA) approach have supported the discriminant validity of the PTSD versus CPTSD distinction (Brewin et al., 2017). These studies have identified distinct classes of trauma-exposed persons characterized by symptom profiles consistent with PTSD and CPTSD, where the latter is comprised of all six symptom clusters organized under the PTSD and DSO factors and the former is comprised of only PTSD symptoms and low DSO symptoms (e.g. Cloitre et al., 2013; Elklit, Hyland, & Shevlin, 2014). Notably, some studies have also found support for an additional class of trauma-exposed individuals who exhibit high levels of DSO symptoms, but low levels of PTSD symptoms (Knefel et al., 2015; Perkonigg et al., 2016), which may represent individuals with other disorders such as depression and dissociative identity disorder that are common in traumatized populations. Finally, confirmatory factor analysis (CFA) studies have found that CPTSD is comprised of two second-order factors, PTSD and DSO, and each of these is measured by the three symptom clusters described above (e.g. Hyland, Murphy, Shevlin et al., 2017; Shevlin et al., 2017). Such findings support the construct validity of the proposed PTSD and CPTSD diagnoses and the characterization of CPTSD as being composed of two factors: PTSD and DSO symptomatology.

A specific measure of ICD-11 PTSD and DSO symptoms has recently been developed: the International Trauma Questionnaire (ITQ; Cloitre, Roberts, Bisson, & Brewin, 2016). Early findings show that the English language version of the ITQ possesses good psychometric properties (e.g. Hyland, Shelvin, Brewin et al., 2017; Karatzias et al., 2016). Given that the ICD-11 PTSD and CPTSD diagnoses are intended to be applicable across different cultures and nations, it is important that the construct and its measurement be evaluated across diverse populations and languages.

The study of ICD-11 PTSD and CPTSD is of particular interest in Lithuania as the study of the psychological impact of trauma is relatively new, coinciding with its independence from the Soviet Union in the 1990s (Kazlauskas & Zelviene, 2016). Studies thus far suggest a population exposed to decades-long pervasive and systematic political oppression and violence during Soviet times (Kazlauskas & Zelviene, 2016). This includes experiences of forced displacement to remote regions of Northern Siberia, political imprisonment, abusive use of psychiatry, and other forms of repression (Kazlauskas, Gailiene, Vaskeliene, & Skeryte-Kazlauskiene, 2017). However, with a high prevalence of trauma in society, PTSD is not acknowledged in health care in Lithuania as it was evidenced by the recent analysis of the National health care registry in Lithuania (Kazlauskas, Zelviene, & Eimontas, 2017).

The aim of this study was to assess the factorial and discriminant validity of the symptoms and structure of CPTSD in a Lithuanian treatment-seeking sample as measured by the ITQ.

## 1. Method

### 1.1. Participants and procedure

The study was approved by the Institutional Psychological Research Ethics Committee. Prior to assessments, each participant was given an oral and written briefing about the study and written informed consent was obtained. Inclusion criteria for this study were: (1) ≥ 18 years old; (2) exposure to at least one lifetime traumatic experience, (3) full completion of the study assessments, and (4) currently in treatment or seeking treatment for mental health problems.

Participants of the study were recruited at primary mental health centres, outpatient mental health clinics and hospitals, private clinical psychologists’ practice, and addiction rehabilitation centres across Lithuania during the period between November 2016 and April 2017. Data was collected in 20 recruitment sites. Participants were interviewed by 20 clinical psychologists, and three clinical psychology master programme students under supervision. Initially, 429 participants were invited to participate in the study of whom 348 participants (81.1%) agreed to take part in the study. A total of 68 participants were excluded from further data analysis because of the following reasons: (1) no trauma exposure (*n* = 29), and (2) did not complete the ITQ assessments (*n* = 39). Excluded participants did not significantly differ in age (*t*(321) = 1.04, *p* = .297), education (χ^2^(5) = 4.17, *p* = .525), and gender (χ^2^(1) = 1.03, *p* = .310) from included participants. In total, 280 participants, 63 men (22.5%) and 217 women (77.5%), were included in this study. Participants’ age ranged from 18 to 84 years, and the mean age was 39.48 (*SD* = 13.35). Demographic characteristics of participants are presented in Table 1.

### 1.2. Measures

We used the ITQ version 1.5.1 (formerly the ICD-Trauma Questionnaire; ICD-TQ) (Cloitre et al., 2016) for PTSD and DSO symptom assessments. The ITQ is based on the WHO ICD-11 proposals for PTSD and CPTSD diagnosis (Maercker et al., 2013). The ITQ was translated into Lithuanian and double back-translated with review by the authors of the original measure.

The ITQ is comprised of 23 symptom items. The three PTSD symptom clusters are assessed with seven items as follows: (1) Re-experiencing (Re) via three items, (2) Avoidance (Av) via two items, and (3) Sense of threat (Th) via two items. The three symptom clusters of DSO are measured with 16 items: nine items for affective dysregulation (AD), four items for negative self-concept (NSC), and three items for disturbances in relationships (DR). For PTSD symptom assessments, participants were asked to rate on a Likert scale from 0 (= *Not at all*) to 5 (= *Extremely*) how much have they been bothered by each of the symptoms during the past month. For DSO assessment, participants were asked to rate how true each statement was of how they typically feel, think about themselves, and relate to others. Reliability of the ITQ measured with Cronbach’s alpha in our sample was good (α = .93). Cronbach’s alpha for PTSD symptoms was α = .88 and for DSO symptoms α = .93.

The presence of the PTSD and DSO symptoms were computed for all participants following the instructions of the authors of the ITQ (Cloitre et al., 2016). PTSD symptoms were coded as positive if the score for at least one of each symptom cluster items was ≥ 2. DSO symptoms were coded as positive if it exceeded half of the total possible sum of all the items at each DSO cluster: the AD was positive for a score of ≥ 10 on five items measuring emotional hyper-activation or a score of ≥ 8 on four items of hypo-activation; NSC symptoms were coded as positive for a score ≥ 8 on four items, and DR symptoms were positive for a score ≥ 6 on three items.

The Life Events Checklist (LEC) (Weathers et al., 2013) consists of 17 items inquiring about lifetime exposure to traumatic events such as physical or sexual violence, combat, captivity or exposure to death or injury, sudden death of a loved one, etc., and the type of exposure (‘happened to me’, ‘witnessed it’, ‘learned about it’, ‘not sure’, ‘does not apply’). Participants were considered exposed to traumatic event if they have reported that they experienced the event or witnessed it. For traumatic events of sudden accidental death and sudden violent death, participants learning about it happening to someone else were also considered to be exposed to a traumatic event. Previous studies of the psychometric properties of the LEC indicated adequate stability, good convergence with other traumatic life events measures, and significant association with PTSD symptoms (Gray, Litz, Hsu, & Lombardo, 2004).

### 1.3. Data analysis

**Figure 1.**
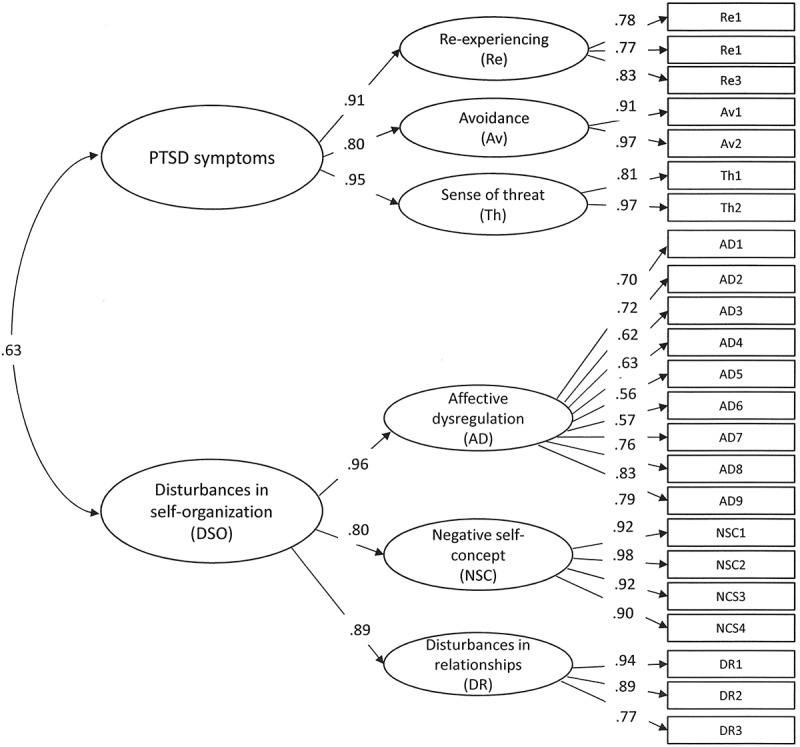
Confirmatory factor analysis of the PTSD and DSO symptoms that comprise ICD-11 complex PTSD. All parameters in the model are significant at *p* < .001.

**Figure 2.**
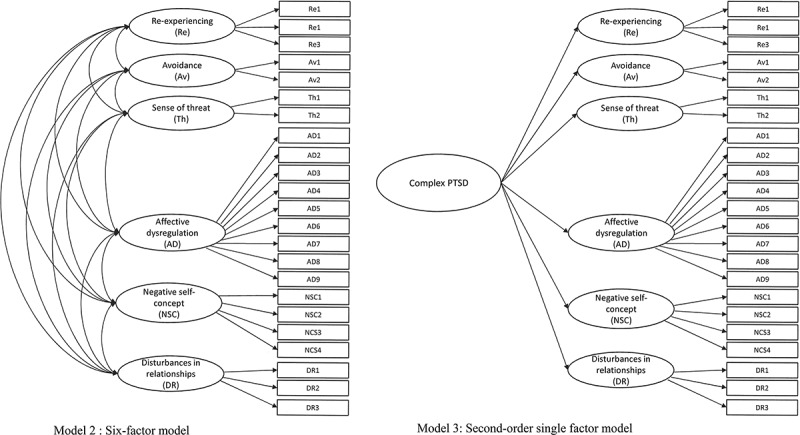
Confirmatory factor analysis of the alternative models of PTSD and DSO symptoms.

Analyses were conducted using Mplus version 6.0. We used CFA with the robust weighted least squares estimator (WLSMV) for structure validity (Flora & Curran, 2004). Three models showing good model fit results in previous studies (Hyland et al., 2017; Karatzias et al., 2016) were tested. The first model was a second-order model of PTSD, as proposed for ICD-11. The second-order PTSD latent factor accounted for the covariation between the Re, Av, and Th factors and the second-order DSO factor accounted for the covariation between the AD, NSC, and DR factors (see Figure 1). This model represented hierarchical structure of CPTSD and distinction between two dimensions of PTSD and DSO. The second CFA model tested was a first-order variant of Model 1 where the second-order factors were omitted. This model represented no distinction between PTSD and CPTSD and no hierarchical structure, where latent factor would explain relationship between symptoms. The third CFA model explained the covariation between the six first-order factors in terms of one second-order latent factor termed ‘Complex PTSD’ (see Figure 2). This model represented hierarchical structure of the symptoms, but no distinction between PTSD and CPTSD was included. All the ITQ items used for CFA analysis were transformed to binary indicators. ITQ items with scores ≥ 2 were coded as ‘1’ and items < 2 were coded as ‘0’. The fit of the CFA models was assessed using the chi-square test, the comparative fit index (CFI), Tucker Lewis index (TLI), and root mean-square error of approximation (RMSEA) indices. CFI and TLI values above .90 indicate acceptable model fit and values above .95 indicate excellent model fit, while RMSEA values of .08 and below indicate acceptable fit and values of .05 and lower indicate good model fit (Kline, 2011).

LCA was used for identification of patient symptom profiles to test if separate classes of individuals having PTSD and CPTSD symptom profiles were distinguishable in accordance with ICD-11 proposals. Binary variables based on cut-offs were calculated for six symptoms clusters of PTSD and CPTSD. The optimal number of classes was evaluated using several fit indices: the bootstrap likelihood ratio test (BLRT), the Lo-Mendell-Rubin adjusted likelihood ratio test (LMR-A), the Akaike Information Criterion (AIC), and the Bayesian Information Criterion (BIC) (Nylund, Asparouhov, & Muthén, 2007). According to the AIC and BIC, the class solution with the lowest value is regarded as the optimal class solution. For the LMR-A and the BLRT, a non-significant value (*p* > .05) indicates that the model with one less class should be accepted.

## 2. Results

### 2.1. Prevalence of traumatic experiences in the sample

Participants experienced on average 4.60 (*SD *= 2.55) lifetime traumatic experiences, ranging from one to 12 events. Exposure to 1–2 traumatic experiences were reported by 22.5% (*n* = 63) of participants, 3–5 traumatic experiences were experienced by 47.5% (*n* = 133) of participants, 6–8 experiences were reported by 21.8% (*n* = 61) of participants, and 9–12 experiences were reported by 8.2% (*n* = 23) of participants. The most common traumatic experiences in the sample were: sudden unexpected death of someone close (69.8%), severe human suffering (54.9%), physical assault (51.8%), car accident (48.4%), childhood physical abuse (39.4%), and sudden violent death (37.7%).

There was a significant, but small gender effect for the total number of lifetime stressors in the sample (*t*(278) = 2.11, *p* = .036, *d* = .30). Women experienced on average 4.43 (*SD* = 2.53) and men experienced 5.19 (*SD* = 2.53) traumatic life events. We found higher prevalence of specific traumatic events among men. Physical assault was experienced by 71.4% of men and 46.0% of women (χ^2^(*df* = 1) = 12.57, *p* < .001), and car accident was experienced by 61.9% men and 44.4% of women (χ^2^(*df* = 1) = 5.95, *p* < .015). There were no significant gender effects on sudden unexpected death of someone close (χ^2^(*df* = 1) = 2.40, *p* = .121), childhood physical abuse (χ^2^(1) = 0.44, *p* = .510) or sexual trauma (χ^2^(1) = 1.85, *p* = .174).

### 2.2. CPTSD structure

Descriptive statistics of the PTSD and DSO symptoms along with symptom inter-correlations are presented in Table 2. The first CFA model with two second-order latent factors of PTSD and DSO showed the best model fit results (χ^2^ (*df* = 223) = 340.360, *p* < .001, RMSEA = .043, CFI = .978, TLI = .975) (see Figure 1). The second CFA model with six correlated factors produced satisfactory but slightly poorer model fit (χ^2^ (*df* = 215) = 338.722, *p* < .001, RMSEA = .045, CFI = .976, TLI = .972). The third CFA model with one second-order factor had the poorest model fit results (χ^2^ (*df* = 224) = 550.097, *p* < .001, RMSEA = .072, CFI = .938, TLI = .930) (see Figure 2). The two-factor, second-order model showed the best fit and is consistent with the theoretical conceptualization of CPTSD as comprised of PTSD and DSO symptomatology, and is therefore viewed as the superior model.

**Table 1.** Demographic characteristics of the sample  (*n* = 280).

Variable	*n*	%
Gender		
Male	63	22.5
Female	217	77.5
Age		
Mean (*SD*)	39.48 (13.35)	–
Range	18–84	–
Relationship status		
In a committed relationship	168	60.4
Not in a committed relationship	108	38.6
Education		
University degree	106	37.9
Some university	19	6.8
Professional college	77	27.5
Finished high school	54	19.3
Obligatory school level or lower	23	8.2
Employment		
Full-time employed	144	51.4
Part-time employed	35	12.5
Not in employment, seeking work	53	18.9
Not in employment, not seeking work	43	15.4
Residence		
Urban	222	79.3
Rural	57	20.4

**Table 2.** Means, standard deviations, symptom prevalence, and ITQ symptoms inter-correlations (*n* = 280).

				Correlations						
International Trauma Questionnaire	*M*	*SD*	*n* (%)	1.	1.1.	1.2.	1.3.	2.	2.1.	2.2.
1. PTSD	1.41	1.03	104 (37.1)	–	–	–	–	–	–	–
1.1. Re-experiencing (3 items)	1.32	1.09	178 (63.6)	.89**	–	–	–	–	–	–
1.2. Avoidance (2 items)	1.53	1.34	152 (54.3)	.83**	.57**	–	–	–	–	–
1.3. Sense of threat (2 items)	1.43	1.21	151 (53.9)	.86**	.69**	.61**	–	–	–	–
2. DSO	1.39	0.87	47 (16.8)	.53**	.45**	.43**	.51**	–	–	–
2.1. Affect dysregulation (9 items)	1.42	0.76	118 (42.1)	.56**	.47**	.46**	.53**	.91**	–	–
2.2. Negative self-concept (4 items)	1.28	1.12	78 (27.9)	.42**	.34**	.35**	.42**	.87**	.69**	–
2.3. Interpersonal disturbances (3 items)	1.26	1.07	81 (28.9)	.42**	.38**	.36**	.35**	.84**	.69**	.63**

ITQ = International Trauma Questionnaire; DSO = Disturbances in self-organization;

** *p* < .01.

The correlations between the factors and the factors loadings of the first CFA model are shown in Figure 1. All first- and second-order factor loadings were positive, statistically significant, and of a robust magnitude. The correlation between the PTSD and DSO factors was moderately strong (*r* = .63).

### 2.3. Latent class analysis

**Figure 3.**
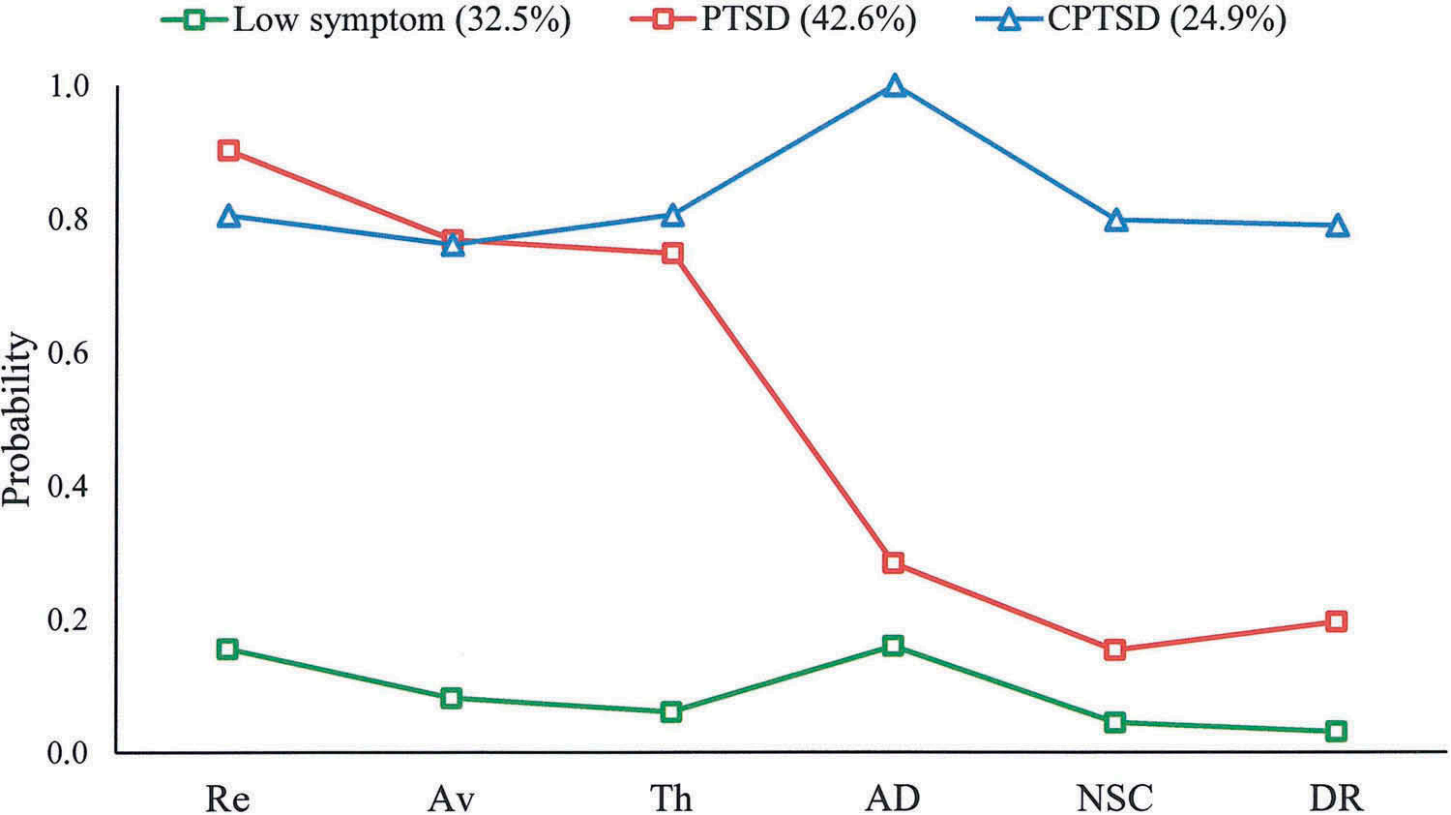
Three latent class models.

The fit statistics for the LCA analyses are reported in Table 3. The results were somewhat ambiguous with the BLRT and the AIC suggesting optimal fit for a four-class solution, and the BIC and LMR-A suggesting optimal fit for a three-class solution. Based on Nylund et al.’s (2007) findings that the BIC is the more reliable indicator of optimal model fit than AIC, we focused our selection of model fit on this index. The profile plots of the three-class solutions are reported in Figure 3.

**Table 3.** Model fit indices of latent class analyses.

Model	Loglikelihood	AIC	BIC	Entropy	BLRT *p*-value	LMR-A *p*-value
2 classes	−957.402	1940.803	1988.055	.792	.000	.000
3 classes	−916.635	1873.270	1945.966	.781	.000	.001
4 classes	−904.559	1863.118	1961.257	.800	.000	.139
5 classes	−900.198	1868.396	1991.979	.738	.500	.499
6 classes	−894.218	1870.436	2019.462	.745	.333	.098

AIC *=* Akaike Information Criterion; BIC = Bayesian Information Criterion; BLRT = Bootstrap Likelihood Ratio Test; LMR-A = Lo-Mendell-Rubin Adjusted Likelihood Ratio Test (LMR-A).

In the three-class solution there is evidence of a ‘CPTSD class’ (24.9%) who exhibit high probabilities of meeting the diagnostic criteria for each of the six CPTSD symptom clusters; a ‘PTSD class’ (42.6%) who exhibit high probabilities of meeting the diagnostic criteria for the three PTSD symptom clusters and low probabilities of meeting the diagnostic criteria for the three DSO symptom clusters; and a ‘Low Symptom’ class who exhibit low probabilities of endorsing each diagnostic criteria (32.5%).

Associations between the three latent classes, demographic data, and lifetime traumatic experiences are presented in Table 4. Individuals in the ‘CPTSD class’ experienced significantly higher levels of sexual trauma, childhood physical abuse, and total number of lifetime traumatic experiences. There was no significant gender or age effect on class membership. Divergent validity of the ITQ was supported with the LCA analysis. The ‘Low symptom’ latent class had no participants with a PTSD or CPTSD diagnosis based on ICD-11 symptom criteria. More than half of individuals in ‘PTSD’ latent class met ICD-11 PTSD diagnostic criteria, and about one third of ‘CPTSD’ latent class individuals met CPTSD diagnostic criteria (see Table 4).

**Table 4.** Characteristics of the three latent classes.

	Latent class			
	Low symptom (*n* = 94)	PTSD (*n* = 109)	CPTSD (*n* = 77)	Significance statistics
Lifetime traumatic experiences, *M* (*SD*)	3.77 (1.94)	4.62 (2.65)	5.58 (2.71)	*F*(2, 277) = 11.64**
Childhood physical abuse	25 (26.6%)	40 (36.7%)	45 (58.4%)	χ^2^(2) = 18.50***
Sexual trauma	10 (10.6%)	20 (18.3%)	27 (35.1%)	χ^2^(1) = 16.02***
ITQ PTSD criteria met	0 (0%)	56 (51.4%)	22 (28.6%)	χ^2^(1) = 66.32***
ITQ CPTSD criteria met	0 (0%)	1 (0.9%)	25 (32.5%)	χ^2^(1) = 67.81***
Gender				
Male	18 (19.1%)	23 (21.1%)	22 (28.6%)	χ^2^(1) = 2.36
Female	76 (80.9%)	86 (78.9%)	55 (71.4%)	
Age, *M* (*SD*)	38.35 (13.62)	40.15 (13.24)	39.83 (13.21)	*F*(2, 256) = .45

ITQ = International Trauma Questionnaire;

** *p* < .01; *** *p* < .001.

## 3. Discussion

This was the first study to test the factorial and discriminant validity of the ICD-11 PTSD and CPTSD proposals in Lithuania, and findings were in line with the theoretical proposals (Maercker et al., 2013). Our findings corroborate earlier validation findings in culturally diverse samples (Elklit et al., 2014; Hyland et al., 2017; Karatzias et al., 2017; Powers et al., 2017; Shevlin et al., 2017). Furthermore, our study supported the factorial and discriminant validity of PTSD and CPTSD in a unique clinical sample of a psychiatric patients, using the newly developed measure for the assessment of ICD-11 PTSD and DSO symptoms (Cloitre et al., 2016; Hyland, Shelvin, Brewin et al., 2017; Karatzias et al., 2016).

The CFA results revealed that the first model with two second-order factors of CPTSD which distinguishes between PTSD and DSO symptomatology provided optimal model fit in this Lithuanian clinical sample. This first model provided similar fit to the second model but was preferred on the grounds of theoretical parsimony. However, the second model with the correlated six-factors distinguishing between PTSD and DSO symptoms at the first-order level continues to offer a viable solution to the structure of CPTSD. Notably, the third model, which does not discriminate between PTSD and DSO symptoms, provided poorer model fit than the models that did acknowledge this distinction. Current findings are therefore consistent with the ICD-11’s proposals for a meaningful difference between PTSD and CPTSD. Additionally, the current study was one of the first to assess the structure of ICD-11 CPTSD symptoms using the ITQ (Cloitre et al., 2016). Current finding add support to the psychometric properties of the ITQ in a previously unstudied cultural sample.

The LCA results provided further support for the discriminant validity of PTSD and CPTSD. Although there was conflicting evidence pointing towards the viability of a three or four class solution, the three-class solution was preferred, since this solution had significant LMR-A and the lowest BIC. Numerous studies have indicated that the BIC is more effective test of optimal fit in LCA than AIC (e.g. Nylund et al., 2007). The LCA results provided evidence of distinct groups of trauma survivors with symptom profiles consistent with PTSD and CPTSD. The symptom profiles identified in the Lithuanian sample are therefore similar to several other studies (Cloitre et al., 2013; Elklit et al., 2014). Furthermore, we found that CPTSD latent class was associated with a higher number of lifetime traumatic experiences, childhood physical abuse, and cumulative sexual trauma similar to other studies (Hyland et al., 2017; Karatzias et al., 2017) and consistent with the CPTSD formulation. Sizable sub-groups in the PTSD and CPTSD latent classes did not meet criteria for PTSD and CPTSD respectively based on the ITQ diagnostic algorithm for ICD-11 PTSD and CPTSD diagnosis in this study. Further studies are needed to improve the ITQ diagnostic criteria in various populations.

While we found promising results supporting the ICD-11 proposals for PTSD/CPTSD in this population, several important limitations of this study ought to be considered. Firstly, data were collected from the general mental health services, mostly among outpatient primary mental health care patients. Generalizability to other samples, including community samples or general population samples, remains unknown. Secondly, the ITQ is a self-report instrument and susceptible to problems of self-report. However, the measure was administered by trained clinicians which alleviates problems of misinterpretation of the meaning of items. Furthermore, even though the ITQ was translated using double back-translation, additional processes to ensure cross-cultural construct equivalence were not taken in our study. Study by De Jong, Komproe, Spinazzola, Van Der Kolk, and Van Ommeren (2005) has shown that these processes might be important in testing CPTSD cross-cultural construct equivalence (De Jong et al., 2005). Moreover, there are currently no available instruments for ICD-11 PTSD or CPTSD diagnosis in the Lithuanian language, therefore cross-validation of our findings with other measures was not possible. However, this was not an epidemiological study, and we focused in this study mostly on validation of the ICD-11 PTSD and CPTSD structure and symptom profile in a clinical sample.

Despite these limitations, the current study contributes to the growing body of knowledge about the factorial and discriminant validity of ICD-11 PTSD and CPTSD. Furthermore, the current findings provide evidence to support the psychometric properties of the ITQ. Finally, this investigation has the potential to contribute to an important specific need which is the systematic assessment and treatment of stress-related disorders in the Lithuanian national health care (Kazlauskas et al., 2017).
